# Suicidal Risk Factors of Recurrent Major Depression in Han Chinese Women

**DOI:** 10.1371/journal.pone.0080030

**Published:** 2013-11-27

**Authors:** Yuzhang Zhu, Hongni Zhang, Shenxun Shi, Jingfang Gao, Youhui Li, Ming Tao, Kerang Zhang, Xumei Wang, Chengge Gao, Lijun Yang, Kan Li, Jianguo Shi, Gang Wang, Lanfen Liu, Jinbei Zhang, Bo Du, Guoqing Jiang, Jianhua Shen, Zhen Zhang, Wei Liang, Jing Sun, Jian Hu, Tiebang Liu, Xueyi Wang, Guodong Miao, Huaqing Meng, Yi Li, Chunmei Hu, Yi Li, Guoping Huang, Gongying Li, Baowei Ha, Hong Deng, Qiyi Mei, Hui Zhong, Shugui Gao, Hong Sang, Yutang Zhang, Xiang Fang, Fengyu Yu, Donglin Yang, Tieqiao Liu, Yunchun Chen, Xiaohong Hong, Wenyuan Wu, Guibing Chen, Min Cai, Yan Song, Jiyang Pan, Jicheng Dong, Runde Pan, Wei Zhang, Zhenming Shen, Zhengrong Liu, Danhua Gu, Xiaoping Wang, Xiaojuan Liu, Qiwen Zhang, Yihan Li, Yiping Chen, Kenneth Seedman Kendler, Jonathan Flint, Ying Liu

**Affiliations:** 1 The First Hospital of China Medical University, Shenyang, Liaoning, P.R.C.; 2 Shanghai Mental Health Center, Shanghai, P.R.C.; 3 Huashan Hospital of Fudan University, Shanghai, P.R.C.; 4 Chinese Traditional Hospital of Zhejiang, Zhejiang, P.R.C.; 5 No. 1 Hospital of Zhengzhou University, Henan, P.R.C.; 6 Xinhua Hospital of Zhejiang Province, Zhejiang, P.R.C.; 7 No. 1 Hospital of Shanxi Medical University, Taiyuan, Shanxi, P.R.C.; 8 ShengJing Hospital of China Medical University, Shenyang, Liaoning, P.R.C.; 9 No. 1 Hospital of Medical College of Xian Jiaotong University, Xian, Shaanxi, P.R.C.; 10 Jilin Brain Hospital, Siping, Jilin, P.R.C.; 11 Mental Hospital of Jiangxi Province, Nanchang, Jiangxi, P.R.C.; 12 Xian Mental Health Center, Xian, Shaanxi, P.R.C.; 13 Beijing Anding Hospital of Capital University of Medical Sciences, Beijing, P.R.C.; 14 Shandong Mental Health Center, Jinan, Shandong, P.R.C.; 15 No. 3 Hospital of Sun Yat-sen University, Guangzhou, Guangdong, P.R.C.; 16 Hebei Mental Health Center, Baoding, Hebei, P.R.C.; 17 Chongqing Mental Health Center, Chongqing, P.R.C.; 18 Tianjin Anding Hospital, Tianjin, P.R.C.; 19 No. 4 Hospital of Jiangsu University, Zhenjiang, Jiangsu, P.R.C.; 20 Psychiatric Hospital of Henan Province, Xinxiang, Henan, P.R.C.; 21 Nanjing Brain Hospital, Nanjing, Jiangsu, P.R.C.; 22 Harbin Medical University, Haerbin, Heilongjiang, P.R.C.; 23 Shenzhen Kang Ning Hospital, Shenzhen, Guangdong, P.R.C.; 24 First Hospital of Hebei Medical University, Shijiazhuang, Hebei, P.R.C.; 25 Guangzhou Brain Hospital Guangzhou, Guangdong, P.R.C.; 26 No. 1 Hospital of Chongqing Medical University, Chongqing, P.R.C.; 27 Dalian No. 7 Hospital, Dalian, Liaoning, P.R.C.; 28 No. 3 Hospital of Heilongjiang Province, Beian, Heilongjiang, P.R.C.; 29 Wuhan Mental Health Center, Wuhan, Hubei, P.R.C.; 30 Sichuan Mental Health Center, Mianyang, Sichuan, P.R.C.; 31 Mental Health Institute of Jining Medical College, Jining, Shandong, P.R.C.; 32 Liaocheng No. 4 Hospital, Liaocheng, Shandong, P.R.C.; 33 Mental Health Center of West China Hospital of Sichuan University, Chengdu, Sichuan, P.R.C.; 34 Suzhou Guangji Hospital, Suzhou, Jiangsu, P.R.C.; 35 Anhui Mental Health Center, Hefei, Anhui, P.R.C.; 36 Ningbo Kang Ning Hospital, Ningbo, Zhejiang, P.R.C.; 37 Changchun Mental Hospital, Changchun, Jilin, P.R.C.; 38 No. 2 Hospital of Lanzhou University, Lanzhou, Gansu, P.R.C.; 39 Fuzhou Psychiatric Hospital, Fuzhou, Fujian, P.R.C.; 40 Harbin No. 1 Special Hospital, Haerbin, Heilongjiang, P.R.C.; 41 Jining Psychiatric Hospital, Jining, Shandong, P.R.C.; 42 No. 2 Xiangya Hospital of Zhongnan University, Changsha, Hunan, P.R.C.; 43 Xijing Hospital of No. 4 Military Medical University, Xian, Shaanxi, P.R.C.; 44 Mental Health Center of Shantou University, Shantou, Guangdong, P.R.C.; 45 Tongji University Hospital, Shanghai, P.R.C.; 46 Huaian No. 3 Hospital, Huaian, Jiangsu, P.R.C.; 47 Huzhou No. 3 Hospital, Huzhou, Zhejiang, P.R.C.; 48 Mudanjiang Psychiatric Hospital of Heilongjiang Province, Mudanjiang, Heilongjiang, P.R.C.; 49 No. 1 Hospital of Jinan University, Guangzhou, Guangdong, P.R.C.; 50 Qingdao Mental Health Center, Qingdao, Shandong, P.R.C.; 51 Guangxi Longquanshan Hospital, Liuzhou, P.R.C.; 52 Daqing No. 3 Hospital of Heilongjiang Province, Daqing, Heilongjiang, P.R.C.; 53 Tangshan No. 5 Hospital, Tangshan, Hebei, P.R.C.; 54 Anshan Psychiatric Rehabilitation Hospital, Anshan, Liaoning, P.R.C.; 55 Weihai Mental Health Center, Weihai, Shandong, P.R.C.; 56 Renmin Hospital of Wuhan University, Wuhan, Hubei, P.R.C.; 57 Tianjin First Center Hospital, Tianjin, P.R.C.; 58 Hainan Anning Hospital, Haikou, Hainan, P.R.C.; 59 Wellcome Trust Centre for Human Genetics, Oxford, United Kingdom; 60 Clinical Trial Service Unit, Richard Doll Building, Oxford, United Kingdom; 61 Virginia Institute for Psychiatric and Behavioral Genetics, Department of Psychiatry, Virginia Commonwealth University, Richmond, Virginia, United States of America; Chiba University Center for Forensic Mental Health, Japan

## Abstract

The relationship between suicidality and major depression is complex. Socio- demography, clinical features, comorbidity, clinical symptoms, and stressful life events are important factors influencing suicide in major depression, but these are not well defined. Thus, the aim of the present study was to assess the associations between the above-mentioned factors and suicide ideation, suicide plan, and suicide attempt in 6008 Han Chinese women with recurrent major depression (MD). Patients with any suicidality had significantly more MD symptoms, a significantly greater number of stressful life events, a positive family history of MD, a greater number of episodes, a significant experience of melancholia, and earlier age of onset. Comorbidity with dysthymia, generalized anxiety disorder (GAD), social phobia, and animal phobia was seen in suicidal patients. The present findings indicate that specific factors act to increase the likelihood of suicide in MD. Our results may help improve the clinical assessment of suicide risk in depressed patients, especially for women.

## Introduction

The relationship between suicidality and major depression (MD) is complex. Suicidal ideation is common and appears to be a precondition for suicide attempts among psychiatric patients with MD. Clinical and epidemiologic studies have consistently demonstrated a strong correlation between MD and suicidality, including suicidal ideation, suicidal plan and suicide attempt [1],[2]. For example, the Vantaa Depression Study showed that 58% of all depressed patients had experienced suicidal ideation; among 15% of the total who had attempted suicide, almost all (95%) had prior suicidal ideation [3].Between 43 to 50% of all suicide victims had previously suffered from a depressive disorder [4]. Furthermore, a history of one suicide attempt strongly predicts the risk for future suicide attempts in the setting of a recurrence of MD [5], [6].

Several studies have shown that, among patients with MD, suicidal ideation and attempts are often correlated with a history of other conditions, including personality disorders[7],[8], anxiety disorders [9],[10],[11],panic attacks[12] and alcohol use disorders[13],[14]. Clinical characteristics and symptomatology have been identified to predict the risk of suicide in MD with and without suicide attempt. Marital isolation, screening age, age of onset, and numbers of episodes have been investigated in MD with suicide attempts but have inconsistent results[15],[16],[17],[18],[19],[20].Sense of hopeless, decreased appetite, lack of energy/fatigue, and psychomotor retardation play important roles in suicide in recurrent major depression[13],[21]. But other controversial evidences show that there are no symptom differences between depressed attempters and non attempters [22].

Many studies have shown that stressful life events (SLEs) played a important etiologic role in affective disorders[23],[24],[25],[26]. Few studies have explored the relationship between the SLEs and suicidality in MD. One older study found that among subjects with MD, there was a positive association with a history of more negative life events and suicide attempts [27]. In our previous study of suicidality in the China, Oxford and VCU Experimental Research on Genetic Epidemiology (CONVERGE) study of MD[28], based on 1,970 MD cases, we previously found a significant association between suicide and total number of SLEs. However, we did not examine the association with specific types of SLEs.

In the present study, we report on suicidality in the complete clinical sample from CONVERGE (N = 6,008 cases). We first attempted to replicate the results previously presented, including the associations between suicidal symptoms and socio-demographic features, clinical features, psychiatric comorbidity and depressive symptoms of patients in recurrent MD. Furthermore, we explore the relationship between age, age of onset, and specific types of SLEs and suicide attempts with the goal of helping to improve the clinical assessment of suicide risk in depressed patients.

## Methods

### Subjects

The data for the present study were drawn from the ongoing China, Oxford and VCU Experimental Research on Genetic Epidemiology (CONVERGE) study of major depression (MD). These analyses were based on a total of 6,008 cases recruited from 51 provincial mental health centers and psychiatric departments of general medical hospitals in 40 cities in 21 provinces, and 5,983 controls who were recruited from patients undergoing minor surgical procedures at general hospitals or from local community centers.

All cases were female and had four Han Chinese grandparents and were excluded if they had a pre-existing history of bipolar disorder, any type of psychosis or mental retardation. Cases were aged between 30 and 60, had two or more episodes of MD, with the first episode occurring between 14 and 50 and had not abused drug or alcohol before the first episode of MD. The mean age of cases was 44.43 (standard deviation 8.88).

All subjects were interviewed using a computerized assessment system, which lasted on average two hours for each case. All interviewers were trained by the CONVERGE team for a minimum of one week in the use of the interview. The interview includes assessment of psychopathology, demographic and personal characteristics, and psychosocial functioning. Interviews were tape-recorded and a proportion of them were listened to by the trained editors who provided feedback on the quality of the interviews. The study protocol was approved centrally by the Ethical Review Board of Oxford University (Oxford Tropical Research Ethics Committee) and the ethics committees in all participating hospitals in China. Major psychotic illness was an exclusion criterion, and the large majority of patients were in remission from illness (seen as out-patients). All interviewers were mental health professionals who are well able to judge decisional capacity. The study posed minimal risk (an interview and saliva sample).

### Measures

The diagnoses of depressive (Dysthymia and Major Depressive Disorder) and anxiety disorders (Generalized Anxiety Disorder, Panic Disorder with or without Agoraphobia) were established with the Composite International Diagnostic Interview (CIDI) (WHO lifetime version 2.1; Chinese version), which classifies diagnoses according to the Diagnostic and Statistical Manual of Mental Disorders (DSM-IV) criteria[29].

The assessment of suicidal features consisted of four questions all asked for the time when they had their lifetime worst depressive episode. The first inquired about preoccupation with death with the phrase “think a lot about death.” Responses to this question were not here analyzed. The second asked about “thought a lot about committing suicide.” A positive response to this item is here considered evidence for suicidal ideation. The next item inquired about making a “plan as to how you might do it.” A positive response here was considered evidence for a suicidal plan. If the responded negative to this item, they then skipped to melancholic symptoms. If they responded that they had a suicidal plan, they were then asked “did you attempt suicide?” A positive response was treated here as “suicide attempt.”

The interview was originally translated into Mandarin by a team of psychiatrists in Shanghai Mental Health Centre with the translation reviewed and modified by members of the CONVERGE team. Phobias, divided into five subtypes (animal, situational, social and blood-injury, and agoraphobia), were diagnosed using an adaptation of DSM-III criteria requiring one or more unreasonable fears, including fears of different animals, social phobia and agoraphobia that objectively interfered with the respondent's life. The section on the assessment of phobias was translated by the CONVERGE team from the interview used in the Virginia Adult Twin Study of Psychiatric and Substance Use Disorders (VATSPUD) [30].

Additional information using instruments employed from VATSPSUD, translated and reviewed for accuracy by members of the CONVERGE team. Information on postnatal depression was assessed using an adaptation of the Edinburgh Scale[31]. The stressful life events section, also developed for the VATSPSUD study, assessed 16 traumatic lifetime events and the age at their occurrence. The childhood sexual abuse was a shortened version of the detailed module used in the VATSPSUD study, which was in turn based on the instrument developed by Martin et al[32]. Neuroticism was measured with the 23-item Eysenck Personality Questionnaire[33], which was also an established instrument for measuring neuroticism.

The case interview was fully computerized into a bilingual system of Mandarin and English developed in house in Oxford, and called SysQ. Skip patterns were built into SysQ. Interviews were administered by trained interviewers and entered offline in real time onto SysQ, which was installed in the laptops. Once an interview was completed, a backup file containing all the previously entered interview data could be generated with database compatible format. The backup file, together with an audio recording of the entire interview, was uploaded to a designated server currently maintained in Beijing by a service provider. All the uploaded files in the Beijing server were then transferred to an Oxford server quarterly.

### Statistical analysis

Socio-demographic and clinical characteristics of the sample were analyzed. For continuous variables, independent Student's t-tests were performed and, for categorical variables, Pearson's chi-squares were calculated. All the characteristics of individuals with suicidality vs non-suicidality MD were assessed by logistic regression in MD, with suicidality as the dependent variable (0 =  absence and 1 =  presence). Associations between variables were expressed as odds ratios (OR) and 95% confidence intervals (95% CI). The associations between suicide attempt and types of SLEs, the difference of the results drawn from previous and current studies were assessed by binary regression. SPSS 18.0 for Windows was used in data analysis. Continuous measures like neuroticism were standardized prior to analysis so that ORs reflect the change in the dependent variable per SD change in the predictor variable. All tests were two-tailed and significance level was defined as 0.05. The homogeneity of the ORs estimated from the first 1,970 and second 4,038 CONVERGE subjects was evaluated by the Breslow-Day Test [34].

Two different approaches might be used for the comparison group for our cases with various levels of severity of suicidal symptomatology. One approach would be to only use the MD cases with no suicidal symptoms of any sort. This would maximize the observed differences. The alternative approach would be to utilize all depressive cases that did not report the severity of the suicidal symptoms being examined. Thus, when examining cases with suicide attempt, the latter approach would include in the comparison group patients with suicidal ideation or plans but not attempts while the former approach would eliminate them. In these analyses, we adopted the latter more conservative approach.

## Results

### Socio-demographic Factors

Data on socio-demographic characteristics of our cases with and without ideation, plan or attempts are seen in table 1. As seen in table 1, there was no significant difference between result of the two independent samples (old data and new data), which showed the samples from the previous[28] and the current were homogeneous.

**Table 1:** Relationship between socio-demographic features of major depression and suicidality.

	No suicidal ideation New (OLD) *Combined*	Suicidal ideation New (OLD) *Combined*	P of difference	Combined P	No suicidal plan New (OLD) *Combined*	Suicidal plan New (OLD) *Combined*	P of difference	Combined P	No attempted suicide New (OLD) *Combined*	Attempted suicide New (OLD) *Combined*	P of difference	Combined P
**Age** a	**43,40±9.07 (44.90±9.01) ** ***43.91±9.10***	**44.66±8.79 (45.15±8.72) ** ***44.87±8.81***	**NS**	**0.000**	**44.16±9.00 (45.16±9.08) ** ***44.52±9.07***	**44.17±8.33 (44.78±8.60) ** ***44.39±8.81***	**NS**	**0.573**	**44.51±8.82 (44.94±8.43) ** ***44.67±8.75***	**43.83±8.83 (44.74±8.85) ** ***44.15±8.88***	**NS**	**0.118**
**Marital Status(%)**												
**Married**	**83.7 (84.1) ** ***83.5***	**83.4 (83.3) ** ***83.6***	**NS**	**0.042**	**84.8 (85.5) ** ***84.9***	**82.0 (81.4) ** ***82.0***	**NS**	**0.004**	**84.7 (85.3) ** ***84.9***	**79.3 (78.5) ** ***79.1***	**NS**	**0.000**
**Never married**	**3.9 (1.7) ** ***3.8***	**3.2 (3.7) ** ***2.8***			**3.3 (2.9) ** ***3.2***	**3.7 (1.8) ** ***3.2***			**3.1 (2.5) ** ***2.9***	**5.0 (2.4) ** ***4.2***		
**Separated/Divorced/Widowed**	**12.4 (14.2) ** ***12.7***	**13.4 (13.1) ** ***13.6***			**12.0 (11.0) ** ***11.9***	**14.2 (16.7) ** ***14.9***			**12.2 (12.7) ** ***12.3***	**15.7 (19.1) ** ***16.7***		
**Education(%)**												
**Primary school/Middle school**	**16.2 (16.3) ** ***16.3***	**23.7 (22.9) ** ***23.5***	**NS**	**0.000**	**20.1 (19.3) ** ***19.9***	**21.5 (21.8) ** ***21.7***	**NS**	**0.001**	**21.0 (20.2) ** ***20.8***	**19.9 (21.1) ** ***20.3***	**NS**	**0.097**
**Technical school**	**52.1 (56.7) ** ***53.4***	**55.6 (55.6) ** ***55.4***			**52.6 (56.1) ** ***53.6***	**56.2 (56.0) ** ***55.9***			**53.3 (56.0) ** ***53.9***	**57.4 (56.2) ** ***57.0***		
**College/graduate**	**31.8 (27.0) ** ***30.3***	**20.8 (21.5) ** ***21.2***			**27.3 (24.7) ** ***26.5***	**22.3 (22.2) ** ***22.5***			**25.7 (23.8) ** ***25.3***	**22.6 (22.7) ** ***22.7***		
**Occupation (%)**												
**Employed**	**38.3 (35.0) ** ***37.3***	**29.0 (29.0) ** ***28.9***	**NS**	**0.000**	**35.6 (33.8) ** ***34.9***	**29.0 (28.0) ** ***28.7***	**NS**	**0.000**	**33.5 (32.5) ** ***33.2***	**29.3 (26.8) ** ***28.5***	**NS**	**0.004**
**Unemployed**	**42.8 (40.9) ** ***42.1***	**52.5 (48.7) ** ***51.2***			**46.1 (42.6) ** ***44.9***	**51.9 (49.9) ** ***51.1***			**47.9 (44.6) ** ***46.7***	**51.7 (50.0) ** ***51.0***		
**Retired**	**18.9 (34.1) ** ***20.6***	**19.5 (22.3) ** ***19.9***			**18.3 (23.7) ** ***20.2***	**19.2 (22.1) ** ***20.2***			**18.6 (22.9) ** ***20.1***	**19.1 (23.2) ** ***20.5***		

This table shows the results of chi square tests for differences in marital status, level of education achieved, and occupational status between those with and without suicidal symptomatology.Data in bracket were from previous research and the italics were from the total data. The combined P were from the total dataP of difference showed the difference between the previous research and present research.

a Age was new-analyzed in this study.

In the combined dataset we found that women with MD and suicide ideation tend to be older than those without ideation (P = 0.000). During their lifetime worst depressive episode, 61.32% (N = 3659) of our cases reported suicidal ideation, 44.61% (n = 2662) reported a suicidal plan and 22.22% (N = 1326) reported a suicide attempt. Female patients without suicidal plan(P = 0.004) and attempt(P = 0.000) were significantly more likely to be married. MD patients with any suicidality (suicidal ideation, plan or attempt suicide) were significantly more likely to be unemployed. Patients with any suicidality had received significantly intermediate education.

### Assessment of clinical features

Table 2 shows the odds ratios from the logistic regression analyses for the association between clinical features of major depression and suicidality. After correcting for the number of comparisons carried, no significant differences (bonferroni p = 0.0013) were found between analyses of the two datasets. [35]. In the combined dateset, patients with any suicidality (suicidal ideation, suicidal plan or attempt suicide had significantly more MD symptoms, and significantly greater number of stressful life events. Patients with suicidal ideation were more inclined to be neurotic, to have experienced childhood sexual abuse and to have a positive family history of MD. Patients with suicidal plan and attempt had significantly more episodes of MD (P = 0.019 and 0.028) and significantly higher rates of melancholia. We noted that an earlier age of onset was associated with higher rates of suicidality (OR = 0.98, P = 0.008).

**Table 2:** Relationship between clinical features of major depression and suicidality.

	Ideation					Plan					Attempt				
	OR New (OLD)	P of difference	Combined OR	Combined CI	Combined P	OR New (OLD)	P of difference	Combined OR	Combined CI	Combined P	OR New (OLD)	P of difference	Combined OR	CombinedCI	Combined P
**Age of onset** a	**1.00 (1.00)**	**NS**	1.00	0.99–1.00	NS	**1.00 (0.99)**	**NS**	**1.00**	**0.99**–**1.00**	**NS**	**0.99 (0.99)**	**NS**	**0.98**	**0.97**–**0.99**	**0.008**
**Number of episodes**	**1.00 (1.00)**	**NS**	1.00	0.99–1.00	NS	**1.01 (1.01)**	**NS**	1.00	1.00–1.01	0.019	**1.01 (1.01)**	**NS**	1.01	1.00–1.01	0.028
**Number of MD symptoms**	**7.98 (9.99)**	**NS**	8.05	6.94–9.34	0.000	**4.72 (4.97)**	**NS**	4.67	4.04–5.41	0.000	**3.22 (3.11)**	**NS**	3.11	2.60–3.73	0.000
**Length of longest episode**	**1.00 (1.00)**	**NS**	1.00	0.99–1.00	NS	**0.99 (1.00)**	**NS**	0.99	0.99–1.00	NS	**1.00 (0.99)**	**NS**	1.00	0.99–1.00	NS
**Neuroticism**	**1.11 (1.03)**	**NS**	1.13	1.04–1.22	0.005	**1.07 (1.01)**	**NS**	1.05	0.98–1.13	NS	**0.91 (0.99)**	**NS**	0.93	0.85–1.00	NS
**Family history of MD**	**1.05 (0.29)**	**NS**	1.06	1.01–1.11	0.011	**1.00 (0.56)**	**NS**	0.99	0.95–1.03	NS	**0.97 (0.53)**	**NS**	0.98	0.94–1.03	NS
**SLE**	**1.08 (1.11)**	**0.036**	1.09	1.03–1.14	0.001	**1.16 (1.25)**	**NS**	1.18	1.03–1.23	0.000	**1.20 (1.25)**	**NS**	1.19	1.14–1.25	0.000
**Melancholia**	**1.05 (1.04)**	**NS**	1.05	0.98–1.11	NS	**1.04 (1.10)**	**NS**	1.07	1.01–1.13	0.030	**1.07 (1.08)**	**NS**	1.08	1.01–1.15	0.018
**Postnatal depression**	**1.04 (1.05)**	**NS**	1.02	0.83–1.26	NS	**1.11 (1.11)**	**NS**	1.06	0.89–1.27	NS	**1.03 (0.99)**	**NS**	0.98	0.80–1.20	NS
**Childhood sexual abuse**	**0.83 (0.77)**	**NS**	0.87	0.77–1.00	0.046	**1.14 (1.30)**	**NS**	1.12	0.99–1.27	NS	**1.06 (1.49)**	**NS**	1.07	0.94–1.21	NS
**PMS**	**0.98 (0.99)**	**0.03**	0.98	0.96–1.01	NS	**0.99 (1.01)**	**0.023**	0.99	0.97–1.02	NS	**1.01 (0.99)**	**NS**	0.99	0.97–1.03	NS

Odds ratios (OR), 95% confidence intervals (CI) and P-values (P) are shown for the relationship between clinical features of major depression (MD) and suicidal ideation, attempt and plan. SLE: stressful life events; PMS: premenstrual syndrome.

Data in bracket were from previous research. The combined were from total data.

P of difference showed the difference between the previous research and present research.

a Age of onset was new-analyzed in this study.

### Assessment of psychiatric comorbidity

Diagnoses of panic disorder, blood phobia were not associated with any suicidality subtype (Table 3). However, cases of MD with any suicidality subtype had significantly higher rates of comorbidity with dysthymia (P<0.05), GAD (P = 0.000), social phobia (P<0.05) (Table 3). We also found significantly higher rates of situational phobia in patients with a suicide ideation and higher rates of agoraphobia in patients with a suicide plan. Animal phobia can be seen in the patients with both suicidal ideation and plan.

**Table 3:** Suicidality as a predictor of major depression with comorbid diseases.

	Ideation					Plan					Attempt				
	OR New (OLD)	P of difference	Combined OR	Combined CI	Combined P	OR New (OLD)	P of difference	Combined OR	Combined CI	Combined P	OR New (OLD)	P of difference	Combined OR	CombinedCI	Combined P
Dysthymia	**1.28 (1.08)**	**NS**	1.28	1.05–1.54	0.012	**1.35 (1.15)**	**NS**	1.30	1.09–1.55	0.004	**1.38 (1.26)**	**NS**	1.33	1.09–1.62	0.004
GAD	**1.53 (1.82)**	**NS**	1.60	1.40–1.82	0.000	**1.27 (1.54)**	**NS**	1.34	1.19–1.51	0.000	**1.23 (1.50)**	**NS**	1.31	1.14–1.51	0.000
Panic disorder	**1.10 (1.05)**	**NS**	1.05	0.84–1.32	**NS**	**1.12 (0.98)**	**NS**	1.06	0.86–1.32	**NS**	**1.06 (0.74)**	**NS**	0.94	0.73–1.21	**NS**
Social phobia	**1.31 (1.45)**	**NS**	1.39	1.13–1.70	0.001	**1.42 (1.43)**	**NS**	1.43	1.19–1.72	0.000	**1.30 (1.17)**	**NS**	1.25	1.01–1.53	0.036
Agoraphobia	**1.39 (0.89)**	**NS**	1.18	0.97–1.43	**NS**	**1.32 (1.11)**	**NS**	1.26	1.05–1.50	0.013	**1.25 (1.20)**	**NS**	1.24	1.01–1.52	0.037
Animal phobia	**1.23 (1.12)**	**NS**	1.20	1.04–1.38	0.016	**1.21 (1.13)**	**NS**	1.19	1.04–1.37	0.014	**1.13 (1.13)**	**NS**	1.14	0.97–1.33	**NS**
Situational phobia	**1.15 (1.35)**	**NS**	1.22	1.02–1.45	0.029	**1.08 (1.39)**	**NS**	1.15	0.97–1.36	**NS**	**0.92 (1.25)**	**NS**	1.05	0.86–1.27	**NS**
Blood phobia	**0.90 (0.78)**	**NS**	0.85	0.72–1.00	**NS**	**0.99 (0.84)**	**NS**	0.91	0.78–1.06	**NS**	**1.12 (0.65)**	**0.02**	0.91	0.75–1.09	**NS**

Odds ratios (OR), 95% confidence intervals (CI) and P-values (P) are shown for the relationship between comorbid anxiety disorders and suicidal ideation, plan and attempt. GAD: generalized anxiety disorder. Data in bracket were from previous research. The combined were from total data.

P of difference showed the difference between the previous research and present research.

### Depressive symptoms and lifetime suicide attempts

The associations between specific depressive symptoms and a reported suicide attempt were explored in table 4. Loss of interest, one of the core diagnostic criteria for MD and insomnia/early morning awakening were associated with suicide attempt in the combined study. The other depressive symptoms most strongly associated with lifetime suicide attempt in women were decreased appetite(OR = 1.46), weight loss (OR = 1.30), insomnia, psychomotor retardation (OR = 1.35), feeling of worthless (OR = 4.18), excessive guilt, diminished concentration and impaired decision-making. Depressed mood, increased appetite, weight gain and psychomotor agitation (OR = 1.20) were significantly associated with suicide attempt during their worst depressive episode.

**Table 4:** Depressive symptoms among women with major depressive disorder and suicide attempt.

Depressive symptom	Adjusted odds ratio (95%CI) New OLD	P of difference	Non suicide attempt	Suicide attempt	Combined Adjusted odds ratio (95%CI)
			Combined N (%)	Combined N (%)	
**Depressed mood**	**1.66(0.87–3.16) 1.28(1.25–1.31)** *	**NS**	**4607(77.7)**	**1324(22.3)**	**1.99(1.16–3.44)** *
**Lost interest**	**1.79 (1.14–2.79)** * **2.56(0.91–7.24)**	**NS**	**4496(77.5)**	**1304(22.5)**	1.79(1.25–2.57)^**^
**Excessive fatigue**	**0.79 (0.39–1.05) 1,13(0.80–1.60)**	**NS**	**4407(78.0)**	**1243(22.0)**	**0.90(0.71–1.14)**
**Decreased appetite**	1.41 (1.14–1.74)^**^1.13(0.80–1.60)^**^	**NS**	**3879(76.9)**	**1167(23.1)**	1.46(1.22–1.74)^**^
**Weight loss**	1. 22 (1.05–1.42)^**^1.32(1.10–1.58)^**^	**NS**	**2730(76.1)**	**858(23.9)**	1.30(1.14–1.47)^**^
**Increased appetite**	**1.19(0.94–1.51) 1.38(1.08–1.76)** *	**NS**	**428(73.7)**	**153(26.3)**	**1.29(1.06–1.57)** *
**Weight gain**	**1.17 (0.88–1.56) 1.32(1.00–1.74)** *	**NS**	**300(73.5)**	**108(26.5)**	**1.29(1.03–1.62)** *
**Insomnia/early morning awakening**	1.86 (1.34–2.59)^**^1.09(0.78–1.51)	**0.021**	**4325(77.4)**	**1264(22.6)**	1. 51 (1.17–1.95)^**^
**Hypersomnia**	**1.15 (0.93–1.41) 0.86(0.66–1.13)**	**NS**	**620(76.7)**	**188(23.3)**	**1.08(0.90–1.29)**
**Psychomotor retardation**	1. 25 (1.13–1.62)^**^1.29(1.05–1.59)^**^	**NS**	**3518(76.7)**	**1070(23.3)**	1.35(1.16–1.57)^**^
**Psychomotor agitation**	**1.16 (0.98–1.37) 1.25(1.02–1.53)** *	**NS**	**3379(77.0)**	**1008(23.0)**	**1.20(1.04–1.38)** *
**Feeling of worthlessness**	3.66(2.79–4.81)^**^4.25(2.86–6.32)^**^	**NS**	**3681(74.6)**	**1255(25.4)**	4.18(3.31–5.26)^**^
**Excessive guilt**	1.73(1.44–2.19)^**^1.80(1.43–2.26)^**^	**NS**	**3357(75.3)**	**1101(24.7)**	1.86(1.60–2.17)^**^
**Diminished concentration**	**1.31 (0.98–1.74) 1.40(1.00–1.96)** *	**NS**	**4279(77.4)**	**1248(22.6)**	1.39 (1.10–1.76) ^**^
**Impaired decision-making**	1.90 (1.50–2.40′)^**^1.71(1.30–2.24)^**^	**NS**	**3847(76.3)**	**1195(23.7)**	1.87(1.55–2.25)^**^

* P<0.05, ** P<0.01, CI confidence interval.

Under data in cell were from preivous research. The combined were from total data.

P of difference showed the difference between the previous research and present research.

### Stressful life events and suicide attempt

Sixteen SLEs were investigated in our study. Patients with suicide attempt reported increased rates of marital separation (OR = 1.51), financial crisis (OR = 1.26), being raped (OR = 1.85) and witnessing an assault (OR = 1.37). Natural disaster (OR = 1.25) and child neglect also were reported in the suicide attempters of women MD. These results are presented in Table 5.

**Table 5:** Relationship between stressful life events and suicide attempt.

Stressful life events^a^	Non suicide attempt	Suicide attempt	Adjusted odds ratio (95%CI)
	N (%)	N (%)	
**Death of a family member**	**885(19.6)**	**269(20.7)**	**1.04(0.88–1.23)**
**Divorce/relationship breakup**	**741(16.4)**	**326(25.1)**	1.51 (1.28–1.77)^**^
**Ever unemployed**	**686(15.2)**	**231(17.8)**	**1.05 (0.87–1.27)**
**Job loss**	**319(7.0)**	**113(8.7)**	**0.98(0.76–1.27)**
**Financial crisis**	**807(17.8)**	**322(24.8)**	1. 26 (1.07–1.48)^**^
**Legal problems**	**154(3.4)**	**61(4.7)**	**0.96(0.69–1.33)**
**Serious illness**	**515(11.4)**	**171(13.2)**	**0.99 (0.81–1.21)**
**Life-threatening accident**	**342(8.0)**	**133(11.0)**	**1.10 (0.88–1.37)**
**Natural disaster**	**431(10.1)**	**169(13.9)**	1.25 (1.03–1.53)^*^
**Witness someone injured**	**321(7.5)**	**142(11.7)**	1. 37 (1.10–1.70)^**^
**Raped**	**61(1.4)**	**47(3.9)**	1.85 (1.23–2.78)^**^
**Physically assaulted**	**277(6.5)**	**127(10.5)**	**1.15(0.90–1.46)**
**Physically abused**	**163(3.8)**	**90(7.4)**	**1.33(0.99–1.79)**
**Seriously neglected**	**400(9.4)**	**184(15.2)**	1.29 (1.04–1.59)^*^
**Threatened**	**741(16.4)**	**326(25.1)**	**0.99(0.61–1.60)**

*P<0.05, ** P<0.01, CI confidence interval.

a Childhood sexual abuse was investigated in independent unit in CONVERGE which can be seen in table 2.

## Discussion

To the best of our knowledge, this is the first study of suicidal symptomatology in Chinese women with MD in a large nationally representative sample. We replicated the results of our previous work and found no significant differences between the two datasets. Our findings can contribute to the better characterization for the suicidal risk of women MD. Our results support the viewpoint that clinical characteristics of depression increased suicidal behavior and the severity of depression was associated with suicidal symptomatology [1],[3].

Several sociodemographic factors were found to be related to suicide in Chinese women with MD in our study. In agreement with other studies, marital isolation increased the risk of suicidal behavior in patients [2], [13]. We also found that unemployment increased the risk of suicide, in agreement with other work [36], [37].

We found systematic differences in the clinical features among women who had versus those who had not attempted suicide. Women with MD and attempted suicide had an earlier age of onset, greater number of episodes, more MD symptoms and more stressful life events. Age of onset is a protective factor in occurrence of suicide attempts in our study, consistent with the result that patients with a history of suicide attempts had an earlier age of onset than non-attempters in recurrent MD[13], the number of episodes, had association with suicide attempts in our study, but was not in others[13],[38].

There were some differences in symptomatic distribution among depressed women who had attempted suicide and those who had not. The strongest relationship with suicide attempt were loss of interest, decreased appetite, weight loss, insomnia/early morning awakening, psychomotor retardation, feeling worthlessness, excessive guilt, diminished concentration and impaired decision-making. Compared with other studies[13],[18],[21],[28], our findings confirmed the importance of feelings of worthlessness in risk assessment, especially given their association with completed suicide [21],[39],[40]. The patients with a history of suicide attempts reported more weight and appetite loss, insomnia, psychomotor activity (agitation or retardation) in our study, in part, supported by other findings [13],[39].Interestingly, we found psychomotor retardation appeared as risk factor for women MD with attempted suicide, however, it was a protective factor against suicide attempts in men[39].

Comorbid illness has also been identified as a risk factor for suicide attempters in MD. [10],[41]. High levels of comorbidity between MD and posttraumatic stress disorder[1],[42], social phobia[11], panic phobia[43] have been reported. In the CONVERGE sample we observed significant associations between suicidality and comorbidity with dysthymia, GAD and social phobia. Further studies with large homogeneous samples should be carried out to identify the association between the comorbid disorder and suicidality in women MD.

Patients with MD reported more stressful life events (SLEs) [24],[26]. Our findings confirmed that the number of depressive symptoms was positively related to the suicide attempts in women MD[28],[39].Inconsistent with our previous study[28],it showed melancholia had typical suicidal relationship in this study. According to other studies, the number of MD symptoms perhaps can be regarded as a stable risk factor for detecting the suicidal behavior in women MD.

To our knowledge, few studies have investigated the relationship between suicide of depression and SLEs for recurrent MD in women, although suicide attempters without depression diagnosis have a higher rate of preceding life events than the depressives [44],[45]. In our study we found marital separation(OR = 1.51), financial crisis(OR = 1.26), witness of assault (OR = 1.37), being raped(OR = 1.85), natural disaster(OR = 1.25) and child neglect(OR = 1.29) may be risk factors for predicting the suicide attempt in depression. This adds to the evidence that depressed patients with suicide attempt have experienced more negative life events[27],[28]. Being raped was the most dangerous stressful life event with near 2 times increased risk of suicide.

In this study several methodological limitations should be considered. First, there was no a continuous quantity index of the severity in suicide. Others have measured the severity of the suicide using a point scale [46]. Second, the time and frequency of occurrence for suicide attempts and SLEs were not considered in our current study. Third, there may be other mediators affecting the relation between the SLEs and suicide of women MD. Because the samples were all women, endocrinal or genetic factors, independent of or additive to the major psychiatric disorders [47], [48], should be explored in the further study of CONVERGE.
